# The impact of early language exposure on the neural system supporting language in deaf and hearing adults

**DOI:** 10.1016/j.neuroimage.2019.116411

**Published:** 2020-04-01

**Authors:** Tae Twomey, Cathy J. Price, Dafydd Waters, Mairéad MacSweeney

**Affiliations:** a Institute of Cognitive Neuroscience, University College London, WC1N 3AZ, UK; b Deafness, Cognition and Language Research Centre, University College London, WC1H 0PD, UK; c Wellcome Centre for Human Neuroimaging, Institute of Neurology, University College London, WC1N 3BG, UK

**Keywords:** fMRI, Age of language acquisition, Sign language, Deaf, Plasticity, Nonsense sign

## Abstract

Deaf late signers provide a unique perspective on the impact of impoverished early language exposure on the neurobiology of language: insights that cannot be gained from research with hearing people alone. Here we contrast the effect of age of sign language acquisition in hearing and congenitally deaf adults to examine the potential impact of impoverished early language exposure on the neural systems supporting a language learnt later in life. We collected fMRI data from deaf and hearing proficient users (N ​= ​52) of British Sign Language (BSL), who learnt BSL either early (native) or late (after the age of 15 years) whilst they watched BSL sentences or strings of meaningless nonsense signs.

There was a main effect of age of sign language acquisition (late ​> ​early) across deaf and hearing signers in the occipital segment of the left intraparietal sulcus. This finding suggests that late learners of sign language may rely on visual processing more than early learners, when processing both linguistic and nonsense sign input – regardless of hearing status. Region-of-interest analyses in the posterior superior temporal cortices (STC) showed an effect of age of sign language acquisition that was specific to deaf signers. In the left posterior STC, activation in response to signed sentences was greater in deaf early signers than deaf late signers. Importantly, responses in the left posterior STC in hearing early and late signers did not differ, and were similar to those observed in deaf early signers. These data lend further support to the argument that robust early language experience, whether signed or spoken, is necessary for left posterior STC to show a ‘native-like’ response to a later learnt language.

## 1 Introduction

In the language literature, ‘age of language acquisition’ effects are typically discussed in the context of second language learning, with the assumption that a robust first language has been established. This situation applies in the vast majority of hearing individuals: only in situations of extreme neglect or severe disability might hearing children not develop a spoken language. In contrast, impoverished early access to language is unfortunately the situation for many children born deaf. Approximately 90–95% of deaf children are born to hearing parents. For these individuals, the first language is typically a spoken language, established on the basis of impoverished auditory input and visual speech (lipreading), which can only afford limited access to the speech signal since many articulators are invisible. The majority of deaf children born to hearing parents are not exposed to sign language in early childhood and often past the point that would normally be considered the sensitive period for language development (see Mayberry and Lock, 2003; Mayberry et al., 2002). Therefore, for many deaf people, unlike hearing people, late sign language acquisition is often related to impoverished exposure to a first language. Here we contrast the impact of age of sign language acquisition in deaf and hearing adults. This allows a unique perspective on the impact of impoverished early language exposure on the neural systems supporting a language learnt later in life.

Two previous studies have examined age of sign language acquisition effects on the neural systems supporting sign language processing in deaf signers (MacSweeney et al., 2008; Mayberry et al., 2011). Differences between early signers and late signers were shown by both MacSweeney et al. (2008) and Mayberry et al. (2011). However, these were not consistent across studies, which used different tasks. Mayberry et al. (2011) reported that activation during a grammatical judgement task and a phonemic-hand judgement task in American Sign Language (ASL) was correlated with age of acquisition positively (i.e. late ​> ​early signers) in occipital regions; and negatively (early ​> ​late) in frontotemporal language regions, including bilateral superior temporal cortices (STC). In contrast, MacSweeney et al. (2008) used a British Sign Language (BSL) phonological judgement task with picture stimuli and reported greater activation in deaf late signers relative to deaf early signers in the posterior part of the left inferior frontal gyrus. There were no regions recruited to a greater extent by deaf early signers than deaf late signers. In both studies, differences between deaf early and late signers could reflect age of sign language acquisition effects and/or the interaction of age of sign language acquisition and deafness. If such effects reflect age of sign language acquisition alone, then the same effects would be expected in a contrast of *hearing* early and late signers. Newman et al. (2002) are the only group to have examined this question. They reported activation in the right angular gyrus during viewing ASL sentences in hearing early signers but not in hearing late signers. No regions were recruited more by hearing late than early signers. Although results in deaf signers from Mayberry et al. (2011) and MacSweeney et al. (2008) differ from each other, neither show increased right angular gyrus activation in early compared to late signers, as reported in hearing signers (Newman et al., 2002). In summary, the existing literature suggests that effects of age of sign language acquisition are different in deaf signers and hearing signers. This discrepancy may be due to differences in the task used or it may indeed reflect the impact of hearing status or a consequence of hearing status: impoverished exposure to a first language, which is related to late sign language acquisition in deaf but not hearing signers.

To fully address the impact of impoverished early language exposure alone on the neural systems supporting language in deaf people, ideally one would manipulate age of BSL acquisition (early/late) and first language experience (full/impoverished). However, by definition, early signers have BSL as their first language and therefore the early signers/impoverished first language group does not exist. In the current study, we therefore used a full factorial design with hearing status (deaf/hearing) and age of BSL acquisition (early/late) as between subject factors. This design allows us to investigate the effects of late sign language acquisition in groups with very different early language experience. We predicted that any observed age of acquisition effects (early vs. late) would differ between hearing signers and deaf signers, possibly reflecting the impact of impoverished early spoken language exposure. It should be noted that the participants recruited in the current study did not learn BSL in adulthood as a *first language* (L1), as reported in studies by Mayberry and colleagues (e.g. Ferjan-Ramirez et al., 2016). Rather the participants had learnt a spoken language as an L1, and BSL as a second language (L2), after the age of 15yrs. Nevertheless, it was the case that all these participants were profoundly deaf from birth and therefore had learnt English on the basis of very impoverished auditory speech input. It is the impact of this impoverished input that is addressed in the current study.

We were particularly interested in age of sign language acquisition effects in superior temporal cortex (STC). In hearing individuals, middle STC primarily responds to auditory input. In deaf people however, parts of STC have been shown to reliably respond to sign stimuli (Capek et al., 2010; Cardin et al., 2013, 2016; MacSweeney et al., 2004) as well as other visual stimuli (Finney et al., 2001; Fine et al., 2005; Vachon et al., 2013; Ding et al., 2015; Bola et al., 2017; Shiell et al., 2014), and this is significantly greater than the response to the same stimuli in hearing signers (e.g., MacSweeney et al., 2002). This difference has typically been interpreted as being driven by some degree of crossmodal plasticity due to hearing status. However, as mentioned earlier, Mayberry et al. (2011) reported a negative correlation in deaf participants between age of ASL acquisition (0–14 years) and activation of the *posterior* superior temporal gyrus (STGs) bilaterally (greater activation in earlier learners) in response to ASL sentences relative to a still image of the signer. It could be argued therefore that the earlier use of a signed language by a deaf child may lead to greater recruitment of the posterior STCs for visual processing, than in a deaf child who learns a signed language later in life. Thus, activation in the posterior part of the STC in response to sign stimuli in deaf signers may reflect the interaction between deafness and early sign language exposure. Alternatively, it could be that the region indentified by Mayberry et al. (2011) is a multisensory region, and also responds to signed input in hearing early signers. A group not tested by Mayberry et al. (2011).

In the current study we investigated the impact of age of sign language acquisition on STC activation in deaf signers and hearing signers in a whole brain analysis and also using the findings of Mayberry et al. (2011) as a region of interest. In addition, we were interested in examining whether any age of sign language acquisition effects in the STCs were observed only for meaningful sign language stimuli or also for nonsense sign sentences. This design allows us to determine whether any effects we observe are due to sign language specific processing or reflects more general activation driven by the visual perception of complex manual actions.

To address these questions we matched deaf and hearing early signers (age of acquisition ​= ​from birth) and late signers (age of acquisition ​> ​15 years) on a BSL grammaticality judgement task (Cormier et al., 2012 ). We then contrasted their BOLD responses while perceiving BSL sentences and nonsense sign sequences. We predicted that any observed age of acquisition effects (early vs. late) would differ between hearing signers and deaf signers, possibly reflecting the impact of impoverished early spoken language exposure. On the basis of the Mayberry et al. (2011) findings we predicted that activation in the posterior STCs would be greater in deaf early signers relative to deaf late signers. Activation patterns in hearing early and late signers would be critical in determining whether this was due to early sign language exposure or early language exposure, regardless of modality. Finally, any interaction between age of sign language acquisition and stimulus type (BSL ​> ​nonsense signs) in the STCs would indicate that any difference between deaf early and deaf late signers may be associated with linguistic processing.

## 2 Materials and methods

### 2.1 Participants

Sixty-two participants were scanned. All participants had learnt BSL, had normal or corrected-to-normal vision and gave informed, written consent to participate in the study, which was approved by the University College London Research Ethics Committee. One participant was excluded due to a data acquisition problem. A further 9 participants were excluded because of poor performance on the experimental task (response sensitivity measured by d’<1.7). See below for which group these participants belonged to. Thus, data from 52 participants were included in the analyses. All participants were right-handed (measured by the Edinburgh inventory; Oldfield, 1971) and without any known neurological abnormality.

Four participant groups were tested: [1] Deaf native signers who learnt BSL from birth from deaf parents (henceforth DE (deaf early); n ​= ​15 (male ​= ​6)); [2] deaf non-native signers who began to learn BSL aged 15 or older (henceforth DL (deaf late), 2 were excluded; n ​= ​11(male ​= ​5) usable datasets); [3] hearing native signers who learnt BSL from birth (henceforth HE (hearing early), 2 were excluded; n ​= ​14 (male ​= ​2) usable datasets); [4] hearing non-native signers who began to learn BSL aged 15 or older (henceforth HL (hearing late), 5 were excluded; n ​= ​12 (male ​= ​6) usable datasets). There were no significant age differences between groups (*F*(3,48)=.922, *p* ​= ​.437, $\omega^{2}$=.000; see Table 1).

**Table 1:** Participant characteristics. Mean [SD] and the range are displayed. The number of participants whose behavioural data were available is indicated (N) as some data were missing.

	Hearing Early	Hearing Late	Deaf Early	Deaf Late
Age (years; months)	N ​= ​14 34; 6 [11; 2] 20; 3–60; 0	N ​= ​12 39; 5 [7; 7] 25; 10–52; 0	N ​= ​15 34; 03 [10; 02] 23; 5–59; 10	N ​= ​11 38; 2 [8; 10] 26; 2–55; 5
Reading comprehension (years; months)	N ​= ​12 17; 8 [1; 10] 14; 8–21; 0	N ​= ​11 20; 5 [1; 11] 15; 8–22; 0	N ​= ​15 16; 2 [1; 10] 13; 6–18; 6	N ​= ​10 17; 0 [2; 5] 13; 0–19; 6
Performance IQ (centile)	N ​= ​14 85 [8] 61–94	N ​= ​12 86 [10] 63–98	N ​= ​15 88 [12] 63–99	N ​= ​11 93 [10] 66–99
English vocabulary (Max ​= ​30)	N ​= ​14 28[2] 24–30	N ​= ​11 28 [2] 26–30	N ​= ​14 27 [2] 23–29	N ​= ​11 27 [2] 22–30
BSL grammaticality judgement (%)	N ​= ​14 80 [8] 67–95	N ​= ​11 84 [6] 73–90	N ​= ​13 83 [10] 67–92	N ​= ​11 85 [5] 77–97
Self-rated BSL skill (1–10	N ​= ​14 8.4 [1.2] 5–10	N ​= ​12 7.6 [1.1] 5–9	N ​= ​14 9.8 [0.4] 9–10	N ​= ​11 8.6[0.5] 8–9
Hearing level in the better ear (dB)	N/A	N/A	N ​= ​8 95 [11] 81–108	N ​= ​5 100 [12] 91–116
Age of BSL acquisition (years; months)	N ​= ​14 00:0 [00:0] 00:0–00:0	N ​= ​12 22; 9 [4; 11] 15; 0–30; 0	N ​= ​15 00:0 [00:0] 00:0–00:0	N ​= ​11 18; 4 [2; 11] 15; 0–25; 0
Duration of sign language experience (years; months)	N ​= ​14 34; 7 [11; 2] 20; 3–60; 0	N ​= ​12 16; 8 [4; 9] 09; 10–28; 0	N ​= ​15 34; 3 [10; 2] 23; 5–59; 10	N ​= ​11 19; 10 [9; 8] 07; 2–39; 5

**Table 2:** Highest level of educational background obtained by deaf participants.

	deaf early signers	deaf late signers
Post-Grad Diploma/PhD	7	6
Degree	3	2
Further Education Teaching Certificate/BTEC/Diploma	3	2
A levels	0	1
Certificate in pre-vocational education	1	0
Data missing	1	0

Participants were tested on a BSL grammaticality judgement task (Cormier et al., 2012), performance IQ (PIQ; block design subtest of the WAIS-R), reading comprehension (Vernon-Warden Reading Comprehension Test, Hedderley, 1996) and English vocabulary (shortened version of the Boston Naming Test, Kaplan et al., 1983). The number of participants whose behavioural data were available is indicated in Table 1 as data were missing from some participants. There were no significant differences among the groups on the BSL grammaticality judgement task (*F*(3,45)=.895, *p* ​= ​.451, $\omega^{2}$=.000) or PIQ (*F*(3,48) ​= ​1.400, *p* ​= ​.254,$\omega^{2}$=.023). Although there was a significant difference across groups on English vocabulary (*F*(3,46) ​= ​2.865, *p* ​= ​.047, $\omega^{2}$=.101), none of the post-hoc tests survived the Bonferroni-corrected statistical threshold. There were group differences on reading attainment (*F(*3,44) ​= ​10.38, *p* ​< ​.001, $\omega^{2}$=.370) such that HL scored significantly better than HE (*t*(21) ​= ​3.316, *p* ​= ​.011, *d*=.479), DE (*t*(24) ​= ​5.424, *p* ​< ​.001, *d*=.783) and DL (*t*(19) ​= ​3.964, *p* ​= ​.002, *d*=.572) (all *p*-values Bonferroni-corrected). There were no significant differences in reading comprehension between the HL, DE and DL groups (*p* ​> ​.05).

All deaf participants reported being born severely or profoundly deaf. Past audiogram data were available for only half of the participants (DE – 8/15; DL – 5/11). The mean hearing loss in the better ear for the DE participants was 95.13 ​dB; range: 81–108. The mean hearing loss in the DL group was 100.0 ​dB; range: 91–116. Fourteen out of 26 hearing participants were BSL interpreters (6/14 HE participants and 8/12 HL participants). See Table 1 for a summary of participant characteristics, including the mean age of BSL acquisition and the mean duration of BSL experience, which were inevitably negatively correlated (r ​= ​-.66, p ​< ​.001).

**Fig. 1:**
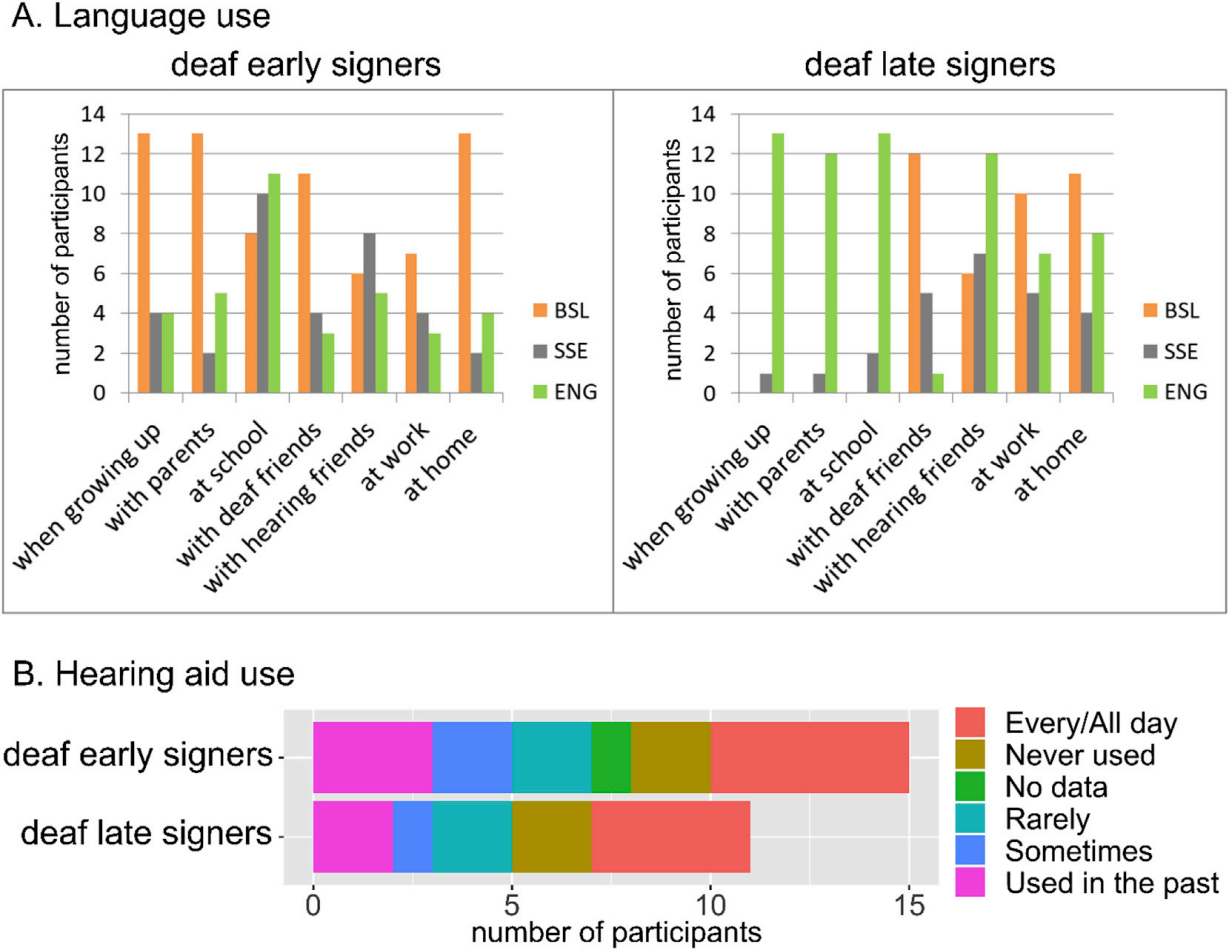
Additional background information on the deaf signers. A: language use in everyday life. BSL = British Sign Language; SSE = Sign Supported English; ENG: spoken English. Many participants selected more than one language in a given situation. B: Hearing aid use.

Given that deaf and hearing people learn a signed language later in life for very different reasons, it is not surprising that deaf late signers learnt BSL significantly earlier than hearing late signers (t(21) ​= ​2.542, p ​= ​.019, d ​= ​1.061). However, deaf and hearing late signers did not differ significantly in their duration of BSL experience (t(21) ​= ​1.010, p ​= ​.324, d=.422). Additional background information of the deaf participants is reported in Fig. 1 (language and hearing aid use) and in Table 2 (education).

### 2.2 Experimental design

Two between-subject factors: hearing status (deaf/hearing) and age of BSL acquisition (early/late); and one within-subject factor, stimulus type (BSL/strings of nonsense signs) were included, resulting in a balanced, 2 ​× ​2 ​× ​2 factorial design.

### 2.3 Stimuli and task

One hundred and twenty full colour video clips, 4 ​s in length, were presented. In each clip, the signer’s hands started from and returned to a rest position in his lap to mark the start and end of each utterance. Sixty of the clips were signed BSL sentences, containing BSL-appropriate mouth and face actions and 60 were nonsense sign sequences. For the signed sentences, participants were required to make a speeded, button response whenever a BSL sentence contained a semantic anomaly. Of the 60 BSL clips, six were ‘yes’ targets containing a semantic anomaly. An example semantic anomaly in English is ‘my favourite colour is a bike’.

During the nonsense sign sequences, participants were required to respond to a target nonsense sign in which the signer placed his left hand in front of his nose with his thumb extended. The 60 nonsense sign sequences consisted of four or five distinct actions, with each action composed of phonotactically legal sub-lexical components (handshapes, movements, locations) of BSL. Of these 60 clips, six featured a target nonsense sign. During the nonsense sign sequences, the signer displayed meaningless face actions similar to those used during the real sentences, with no mouthing. The anomalous word or target nonsense sign appeared towards the end of the sentence or string of nonsense signs. The mean duration was 2525 ​ms (SE ​= ​50) for the BSL sentences and 2975 ​ms (SE ​= ​38) for the nonsense signs. The nonsense sign sequences were longer than the BSL sentences (*t*(118) ​= ​7.235, *p* ​< ​.001, *d* ​= ​1.321). To view example videos, click on the image visible below (online version only).

Supplementary video related to this article can be found at https://doi.org/10.1016/j.neuroimage.2019.116411

The following are the supplementary data related to this article:

Video Video

Video Video

## 3 Procedure

Participants practiced the tasks prior to going into the scanner, with stimuli not included in the experiment. For all participants, the right index finger was used to respond to ‘yes’ trials. ‘No’ trials did not require a response. There was one target sentence/nonsense sign string in each block.

Each participant completed two fMRI runs, each of which lasted 6 ​min. These runs consisted of twelve 21-sec blocks alternating between BSL sentences (six blocks) and nonsense sign sequences (six blocks). One run began with BSL sentences and the other with nonsense sign sequences. The order of the blocks was counterbalanced across participants within group. Video blocks were interspersed with ten 9-sec fixation blocks and two longer 18-sec fixation blocks. Each block began with a 1-sec printed English task prompt: ‘nonsense?’ for the BSL condition or ‘nose?’ for the nonsense sign condition. Five 4-sec video clips followed the task prompt. In each clip, the signer’s hands started from and returned to a rest position in his lap to mark the start and end of each utterance. While each clip (trial) lasted 4 ​s, the duration of the individual BSL sentences or the nonsense sign strings varied between 1707 ​ms and 3486 ​ms, effectively providing jittered inter-stimulus intervals, during which participants saw the signer with his hands on his lap. Stimuli were projected onto a screen positioned at the top of the scanner bore. Participants viewed the stimuli via a mirror placed on the MRI head coil.

### 3.1 MRI acquisition

Anatomical images were acquired from all participants using a Siemens 1.5-T Sonata scanner. Anatomical T1-weighted images were acquired using a 3-D MDEFT (modified driven equilibrium Fourier transform) sequence. One hundred and seventy-six sagittal partitions with an image matrix of 256 ​× ​224 and a final resolution of one mm^3^ were acquired (repetition time (TR): 12.24 msec; echo time (TE): 3.5 msec; inversion time (TI): 530 msec). Functional T2*-weighted echo-planar images with BOLD contrast comprised 38 axial slices of 2 ​mm thickness (1 ​mm gap), with 3 ​× ​3 ​mm in-plane resolution. One hundred and twenty-one volumes were acquired per run (repetition time (TR): 3.42 ​s; echo time (TE): 50 msec; flip angle ​= ​90°). TR and stimulus onset asynchrony were mismatched, allowing for distributed sampling of slice acquisition across the experiment (Veltman et al., 2002), and therefore no need for explicit jittering. To avoid Nyquist ghost artifacts, a generalized (trajectory-based) reconstruction algorithm was used for data processing. After reconstruction, the first six volumes of each session were discarded to ensure tissue steady-state magnetization.

### 3.2 Statistical analysis

The d’ scores and reaction times (RTs) were the dependent measures of behavioral performance. For the calculation of the d’ scores, corrections of ±0.01 were made since some participants had the hit rate of 1 and/or the false alarm rate of zero. RTs were available for ‘yes’ trials only and were recorded from the onset of the stimulus. The RT analysis included correct (target detection) trials only. Behavioral data were analysed in a 2 ​× ​2 ANOVA with hearing status (deaf/hearing) and the age of BSL acquisition (early/late) as between-subject factors; separately for each stimulus type (BSL/nonsense signs).

The imaging data were processed using SPM12 (Wellcome Trust Centre for Neuroimaging, London UK; http://www.fil.ion.ucl.ac.uk/spm/). All functional volumes were spatially realigned and unwarped in order to adjust for minor distortions in the B0 field due to head movement (Andersson et al., 2001). All functional images were normalized to the Montreal Neurological Institute (MNI) space (maintaining the original 3 ​× ​3 ​× ​3 ​mm resolution). Functional images were then smoothed using an isotropic 6 ​mm full-width half-maximum (FWHM) Gaussian kernel.

First-level fixed-effects analyses were based on a least squares regression analysis using the general linear model in each voxel across the whole brain. Low-frequency noise and signal drift were removed from the time series in each voxel with high-pass filtering (1/128 ​Hz cutoff). Residual temporal autocorrelations were approximated by an AR(1) model and removed. At the first level, the onsets of trials (4 ​s video) were modelled as epoch-related responses and convolved with a canonical haemodynamic response function. Correct trials (correct ‘yes’ trials and correct no response trials) for each of the two conditions and the errors over two sessions were modelled separately. Button press manual responses and the task prompts were modelled as event-related responses and convolved with a canonical haemodynamic response function. Fixation was not modelled and served as an implicit baseline. The reaction time data were not used in the imaging analyses since they were only from ‘yes’ trials, which made up only 20% of all trials. The contrasts of interest were: 1) BSL, 2) strings of nonsense signs, 3) BSL ​> ​strings of nonsense signs, 4) BSL and strings of nonsense signs, averaged over sessions.

At the second-level, the contrast images from the first level were used to run random-effects analyses. We investigated main effects of age of BSL acquisition, hearing status and stimulus type; and their interactions using the partitioned error approach. We report activation as significant at voxel-level inference of *p* ​< ​.05, family wise error (FWE) corrected for multiple comparisons at the whole brain level.

In order to investigate whether activation is greater in deaf early than deaf late signers in the part of STC where Mayberry et al. (2011) reported a negative correlation with age of ASL acquisition, we defined two a priori regions-of-interest (ROIs) based on the peak STC coordinates during ASL grammaticality judgement from Mayberry et al. (2011): the left superior temporal gyrus [x ​= ​−42, y ​= ​−36, z ​= ​2] and the right superior temporal gyrus [x ​= ​54, y ​= ​−36, z ​= ​16]. We extracted the eigenvariate values from an 8 ​mm sphere centred on the peak coordinates from i) BSL sentences and ii) nonsense signs. In order to determine whether any observed effect of age of sign language acquisition in the STC ROIs was associated with linguistic processing, we also contrasted the two groups on BSL and strings of nonsense signs.

To compare the results with those in Mayberry et al. (2011), we first report results of the ROI analyses in deaf signers only. To examine whether age of sign language acquisition differentially affects deaf signers and hearing signers, we then directly contrast deaf and hearing signers (age of acquisition ​× ​hearing status ​× ​stimulus type). The statistical analyses were run for the left and right STC ROIs separately, with JASP (JASP Team, 2018).

## 4 Results

### 4.1 Behavioural data

**Fig. 2:**
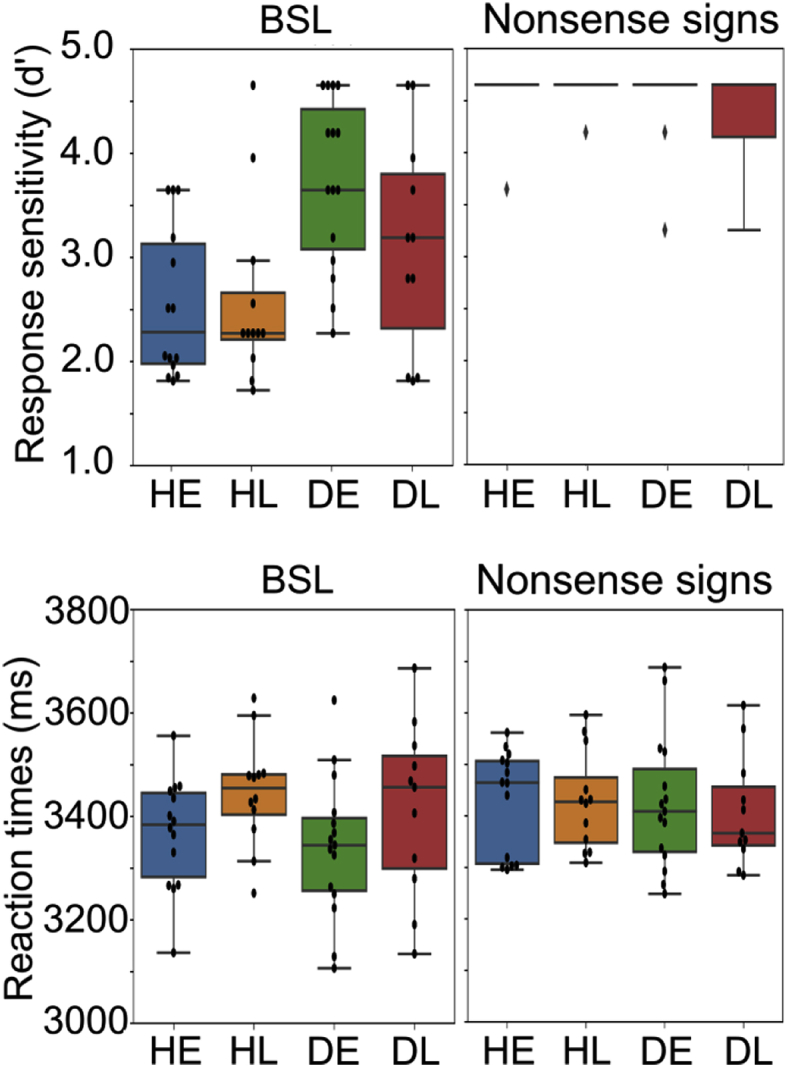
Behavioural data displayed as boxplots. Top row: response sensitivity (d’); BSL on the left, strings of nonsense signs on the right. Bottom row: reaction times (ms); BSL on the left, strings of nonsense signs on the right. Each data point is displayed as a black dot. Accuracy by all groups was at ceiling on the nonsense sign task and there were no significant effects of hearing status, age of BSL acquisition or interactions on reaction times. During the BSL task, response sensitivity was affected by hearing status (deaf ​> ​hearing) whilst reaction time was affected by age of BSL acquisition (early ​< ​late).

The behavioural data are plotted in Fig. 2. Accuracy on the target detection task with the nonsense sign stimuli was very high. The d’ scores for strings of nonsense signs were at the ceiling level. The following numbers of participants in each group scored the highest possible d’ score of 4.65: HE: 13/14; HL: 11/12; DE: 12/15; DL: 8/11 (see Fig. 2). Thus, these data were not analysed further. The analyses of the d’ scores for the BSL task showed that there was a significant main effect of hearing status (*F*(1,48) ​= ​12.685, *p* ​< ​.001, $\omega^{2}$=.180), indicating that deaf signers were better (3.471) than hearing signers (2.568) at identifying the semantically anomalous sentence. However, there was no main effect of age of BSL acquisition (*F*(1,48) ​= ​1.363, *p* ​= ​.249, $\omega^{2}$=.006; early ​= ​3.185, late ​= ​2.846) and no interaction between hearing status and age of acquisition (*F*(1,48) ​= ​1.758, *p* ​= ​.191, $\omega^{2}$=.012).

The reaction time analyses included target detection (correct ‘yes’) trials. For BSL, there was a significant main effect of age of acquisition (*F*(1,48) ​= ​4.340, *p* ​= ​.043, $\omega^{2}$=.062), indicating that early signers were faster (3354 ​ms) than late signers (3431 ​ms). There was no significant effect of hearing status (*F*(1,48)=.654, *p* ​= ​.423, $\omega^{2}$=.000) and no significant interaction between hearing status and age of BSL acquisition (*F*(1,48)=.004, *p* ​= ​.950, $\omega^{2}$=.000). For strings of nonsense signs, there were no significant effects of hearing status (*F*(1,48)=.152, *p* ​= ​.698, $\omega^{2}$=.000), age of acquisition (*F*(1,48)=.071, *p* ​= ​.791, $\omega^{2}$=.000) and no interaction (*F*(1,48)=.081, *p* ​= ​.777, $\omega^{2}$=.000).

In summary, these data suggest no effect of hearing status or age of BSL acquisition on response to nonsense signs. In response to BSL stimuli, the effect of hearing status was evident in response sensitivity (deaf better than hearing) but not in reaction times. In contrast, age of acquisition affected reaction times (early learners quicker than late learners) but not response sensitivity.

### 4.2 Imaging data

#### 4.2.1 Age of BSL acquisition – whole brain analyses

**Fig. 3:**
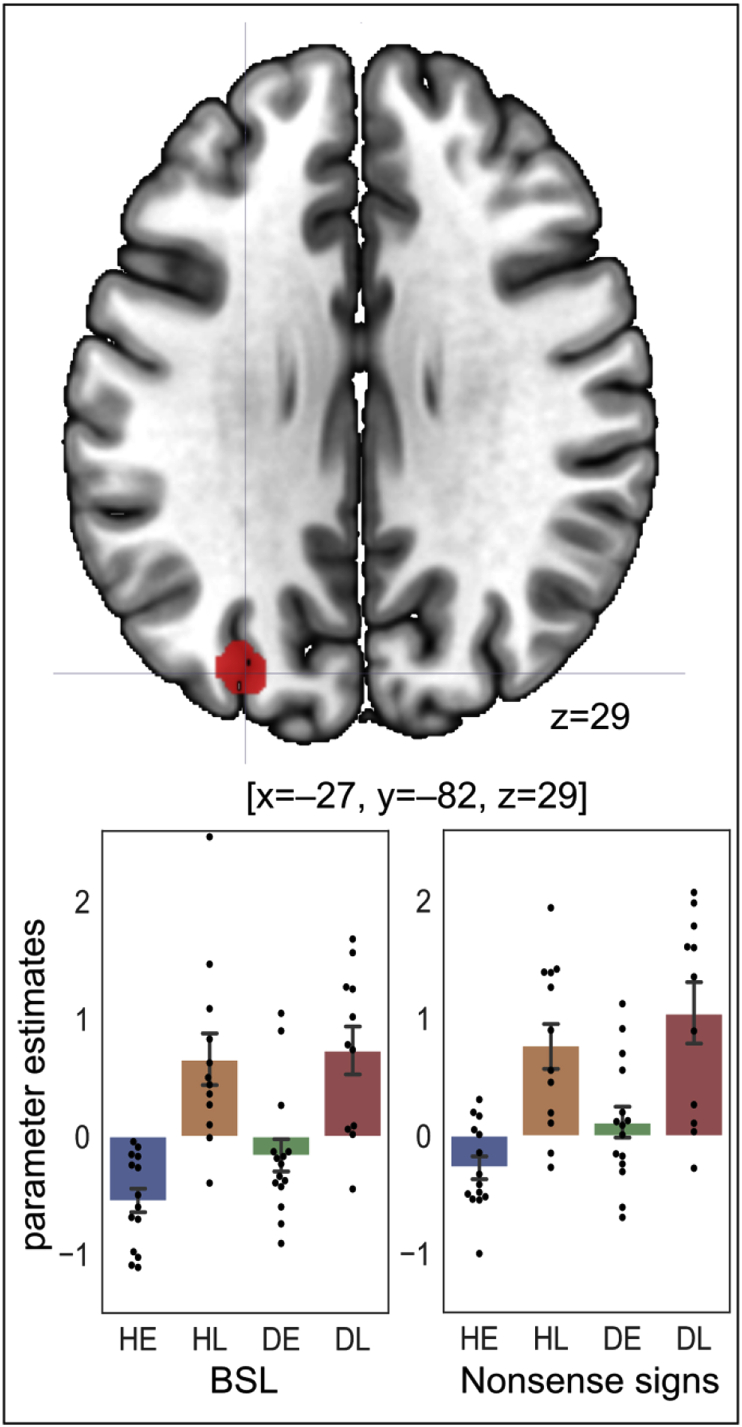
The main effect of age of BSL acquisition (late ​> ​early) in deaf and hearing signers. Top row: the effect is shown at *p* ​< ​.001 uncorrected. This effect was centred on the left paroccipital segment (the occipital end) of the intraparietal sulcus (Petrides, 2011). The crosshair indicates the peak coordinate at [x ​= ​−27, y ​= ​−82, z ​= ​29]. Bottom row: bar plots showing the parameter estimates at this peak for BSL on the left, nonsense sign strings on the right. Each data point is also displayed as a black dot. Error bars indicate standard errors.

There was a significant main effect of age of sign language acquisition at [x ​= ​−27, y ​= ​−82, z ​= ​29; *t*(50) ​= ​6.06, *Z* ​= ​5.19, *p* ​= ​.005 FWE corrected for multiple comparisons] where responses were greater in late (Z ​= ​4.16) than early signers (Z ​= ​−2.24), see Fig. 3. This effect was centred on the left paroccipital segment (the occipital end) of the intraparietal sulcus (Petrides, 2011). At the lower threshold (*p* ​< ​.001 uncorrected & *k* ​> ​10), this effect extended into both middle and superior occipital gyri (*k* ​= ​48). There were no significant effects for the opposite contrast of early ​> ​late, at the corrected level. Nor were there any significant interactions between age of acquisition and hearing status or stimulus type.

### 4.3 Hearing status

**Fig. 4:**
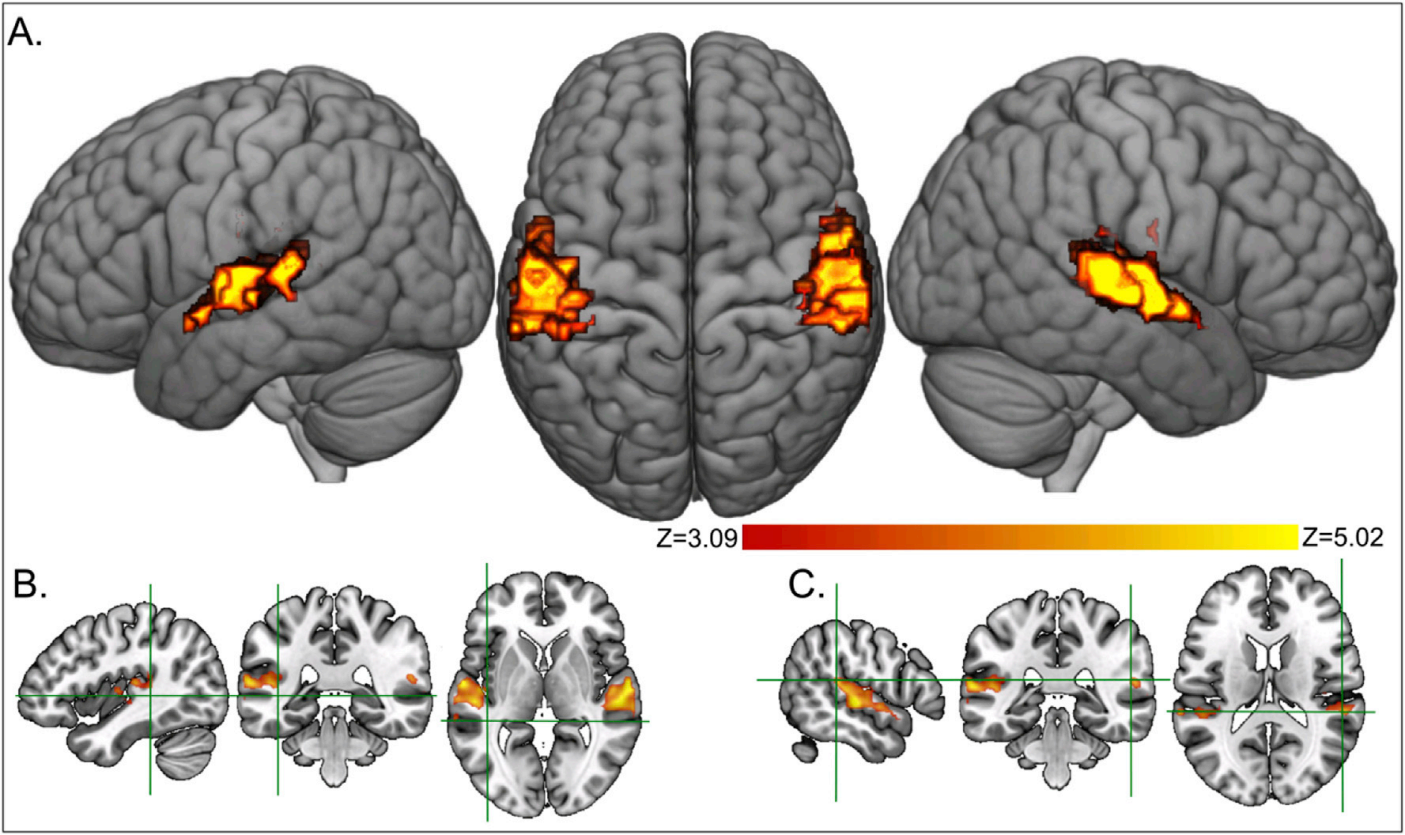
Main effects of hearing status (deaf ​> ​hearing). A: the effects are displayed at *p* ​< ​.001 uncorrected (k ​> ​10), shown in red (Z ​= ​3.09) to yellow (Z ​= ​5.02). The FWE corrected threshold of *p* ​< ​.05 equals to Z ​> ​4.75. The strongest effects in both height and extent were in the middle part of the STC. B & C: the crosshairs show the centre of the ROI (8 ​mm) in the left ROI (B) and the right ROI (C), indicating their relative location to the main effects of hearing status.

Significant main effects of hearing status (deaf ​> ​hearing) at *p* ​< ​.05 FWE corrected were found within large areas of the superior temporal cortices (STCs) bilaterally (Table 3). The effects are displayed at a lower threshold of *p* ​< ​.001 uncorrected (k ​> ​10) in Fig. 4A in red (Z ​= ​3.09; *p* ​< ​.001 uncorrected) to yellow (Z ​= ​5.02). The strongest effects in both height and extent were in the middle part of the STC. The effect in the right STC did not include any voxels within the right STC ROI. The effect in the left STC included just one voxel within the left STC ROI (see Methods). Both STC ROIs were more posterior to the location of the main effect reported here (Fig. 4B & C). The responses in deaf signers were of activation whereas those in hearing signers were mostly of deactivation (see Table 3 for details). The opposite contrast of hearing ​> ​deaf revealed no significant activation at the corrected level.

**Table 3:** Main effects of hearing status (deaf ​> ​hearing) at *p* ​< ​.05 FWE corrected. The largest Z-scores per group relative to the baseline (within the cluster of deaf ​> ​hearing) illustrate that hearing signers mostly deactivated and deaf signers activated relative to baseline. ** ​= ​significant at *p* ​< ​.05 FWE corrected; * ​= ​significant at *p* ​< ​.001 uncorrected.

Deaf ​> ​hearing at *p* ​< ​.05 FWE corrected											Each group relative to baseline																												
											HE							HL							DE							DL							
k	Regions	x	y		z		Z		p		x	y		z		Z		x	y		z		Z		x	y		z		Z		x		y		z		Z	
Left STC																																							
2	Heschl’s gyrus	−45		−13		5		5.31**		0.003	−45		−13		5		−3.83*	−48		−13		5		−4.35*	−45		−13		5		0.98		−45		−13		5		0.88
1	Planum temporale	−45		−34		8		4.81**		0.037	−45		−34		8		−2.96	−45		−34		8		−0.22	−45		−34		8		4.00*		−45		−34		8		1.34
1	Planum temporale	−54		−31		8		4.78**		0.044	−54		−31		8		−2.27	−54		−31		8		−0.03	−54		−31		8		4.03*		−54		−31		8		2.28
28	Planum temporale	−57		−19		8		5.49**		0.001	−57		−19		8		−3.54*	−57		−13		5		−3.41*	−54		−19		5		3.25*		−57		−16		5		2.60
	Planum temporale	−51		−22		2		5.07**		0.010	−48		−25		5		−2.92	−48		−25		5		−1.95	−51		−22		2		3.15*		−51		−22		2		3.95*
	Superior temporal gyrus	−60		−7		2		5.39**		0.002	−60		−7		5		−3.54*	−60		−7		−2		−1.16	−60		−10		5		3.38*		−60		−7		−1		4.02*
1	Superior temporal gyrus	−63		−19		−1		4.78**		0.044	−63		−19		−1		0.30	−63		−19		−1		0.30	−63		−19		−1		3.89*		−63		−19		−1		3.06
4	Superior temporal gyrus	−63		−34		11		5.27**		0.003	−63		−34		11		0.07	−63		−34		11		0.09	−60		−34		−11		4.68*		−60		−34		−11		3.80*

Right STC																																							
4	Planum polare	48		−16		−1		5.45**		0.001	48		−13		−1		−3.67*	48		−13		−1		−3.79*	48		−16		−4		3.05		48		−16		−4		3.52*
39	Superior temporal gyrus	57		−19		−1		5.76**		0.000	63		−10		5		−2.25	63		−13		2		−2.85	54		−25		−1		6.31**		57		−19		−1		4.57*
	Superior temporal gyrus	60		−4		−4		5.32**		0.003	60		−4		−1		−2.13	60		−4		−1		−3.40*	63		−7		−7		4.14*		60		−4		−4		3.24*
	Planum temporale	63		−10		2		5.51**		0.001	63		−10		5		−2.25	63		−7		2		−3.30*	63		−10		2		2.42		63		−7		2		4.49*
7	Planum temporale	63		−22		8		5.03**		0.012	60		−19		8		−2.80	63		−22		8		−2.51	66		−22		8		2.53		66		−25		8		3.77*

### 4.4 Stimulus type

**Fig. 5:**
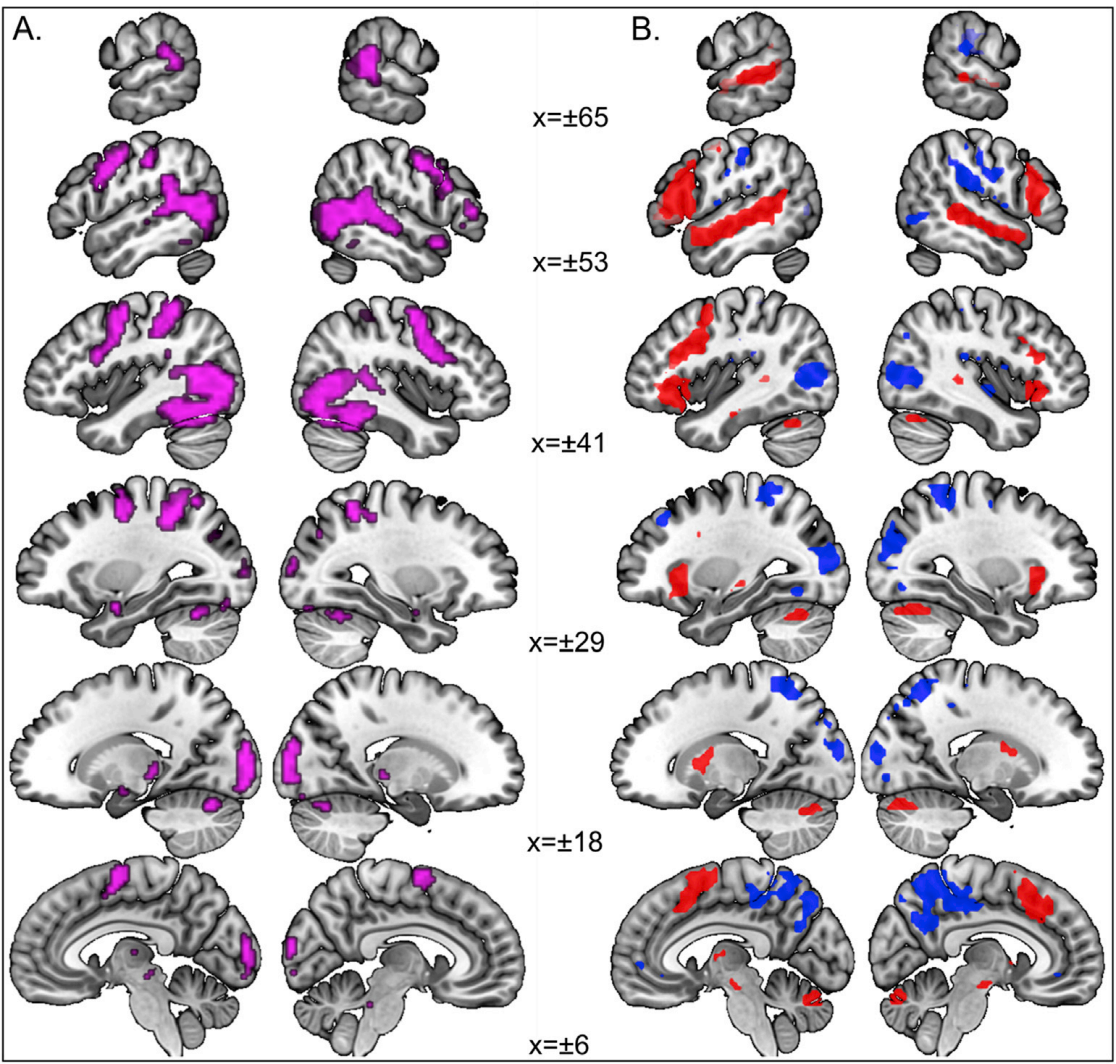
Effects of stimulus type at *p* ​< ​.05 FWE corrected. A) The overlap of [BSL ​> ​rest] and [strings of nonsense signs ​> ​rest] in pink. B) BSL ​> ​strings of nonsense signs in red; Strings of nonsense signs ​> ​BSL in blue. Despite the overlap in the posterior regions (pink in A), some of the effects were significantly greater for nonsense signs than BSL (blue in B).

**Fig. 6:**
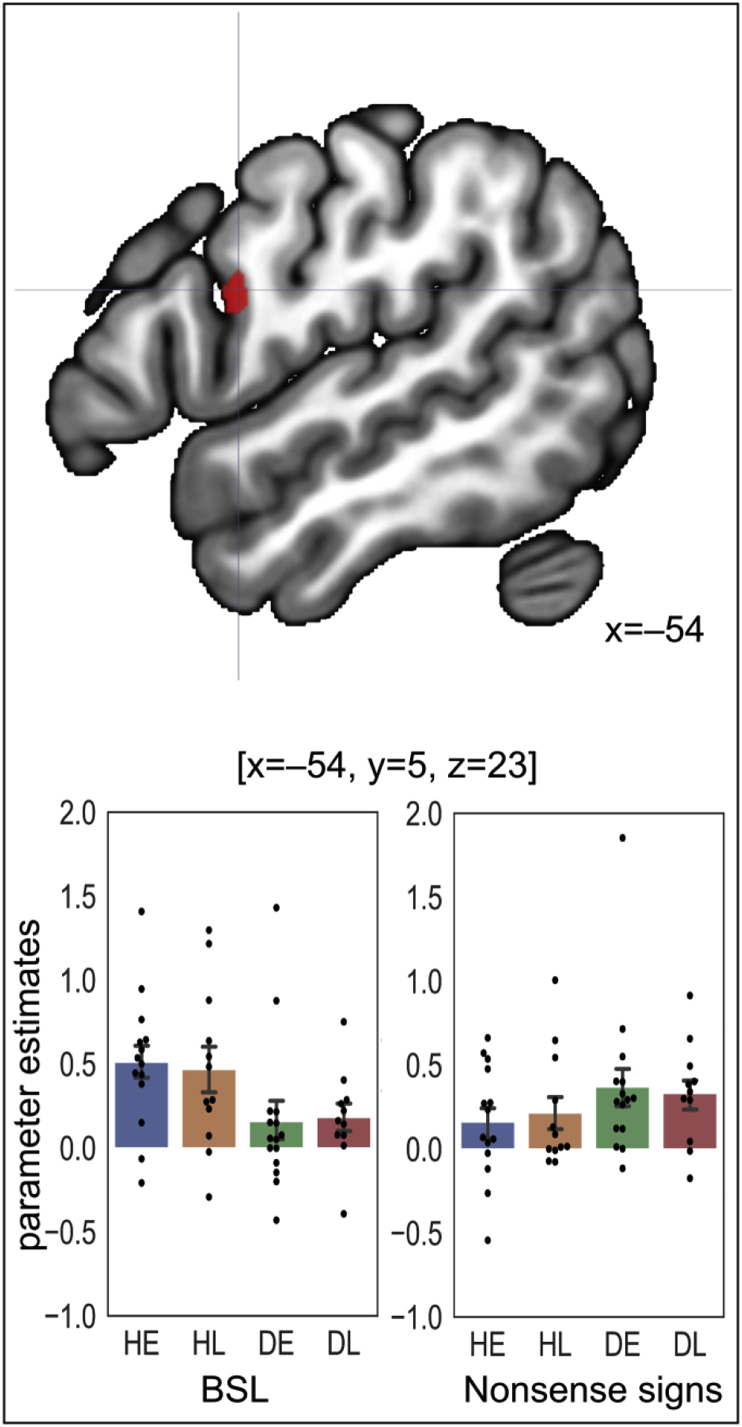
The interaction between hearing status and stimulus type in the left precentral gyrus at [x ​= ​−54, y ​= ​5, z ​= ​23]. Top row: the effect is shown at *p* ​< ​.05 FWE corrected. Bottom row: bar plots showing the parameter estimates at this peak for BSL on the left, strings of nonsense signs on the right. Each data point is also displayed as a black dot. Error bars indicate standard errors.

Activation that was greater for BSL than strings of nonsense signs was mostly in bilateral superior temporal cortex, frontal cortex, as well as caudate, thalamus and cerebellum (Fig. 5B in red). In contrast, activation was greater for strings of nonsense signs than BSL in posterior regions bilaterally, including occipital cortex, precuneus and parietal operculum; and right postcentral gyrus and supramarginal gyrus and posterior middle temporal gyrus (see Fig. 5B in blue). Stimulus type also interacted with hearing status (see Fig. 6). This effect was observed in the left precentral gyrus, at [x ​= ​−54, y ​= ​5, z ​= ​23; *t*(49) ​= ​7.12, *Z* ​= ​5.89, *p* ​< ​.001 FWE corrected, *k* ​= ​9; see Fig. 6) where activation was greater for BSL than nonsense signs in hearing signers (*t*(25) ​= ​5.27, Z ​= ​4.28, significant at *p* ​< ​.001 uncorrected) and greater for nonsense signs than BSL in deaf signers (*t*(25) ​= ​4.88, Z ​= ​4.05, significant at *p* ​< ​.001 uncorrected). However, activation did not differ in this region between deaf and hearing signers for either BSL or strings of nonsense signs due to the greater between-subject variance than the within-subject variance.

### 4.5 Region of interest analysis: age of sign language acquisition effects in STC ROI

**Fig. 7:**
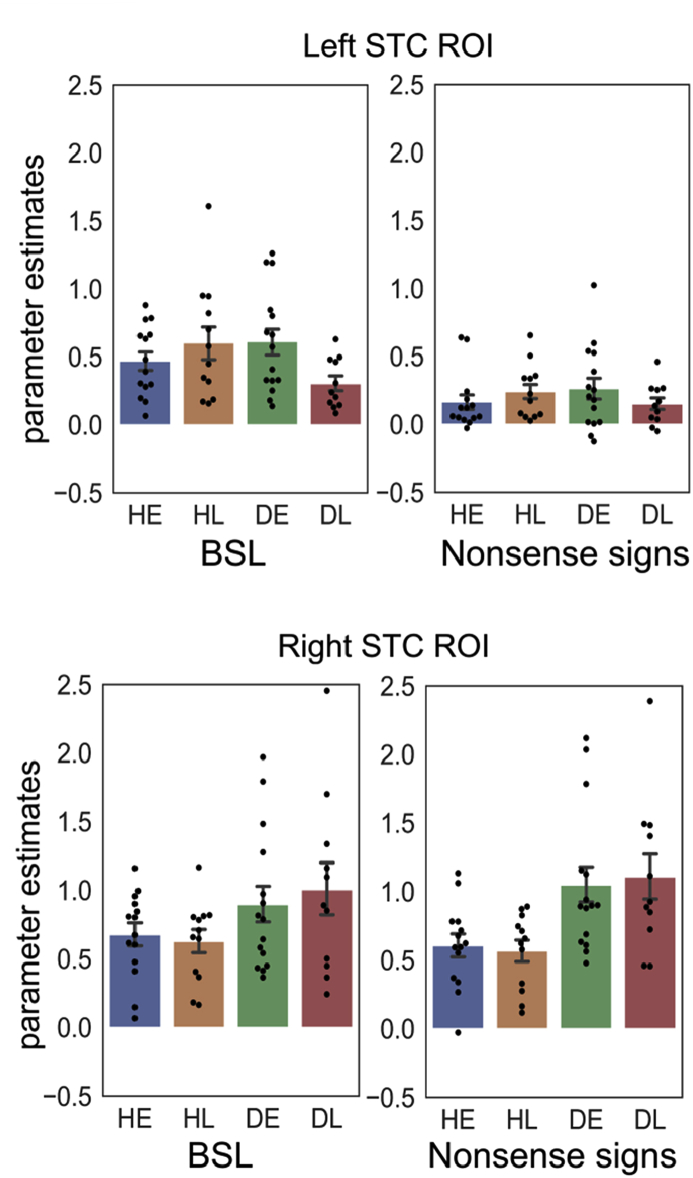
The bar plots of parameter estimates from the ROIs. Left STC on the top; Right STC on the bottom. In the left STC ROI, the effect of age of acquisition was significant in deaf signers only (deaf early ​> ​deaf late); and activation was greater for BSL than strings of nonsense signs across groups. In the right STC ROI, there were no age of acquisition effects in deaf or hearing signers. However, activation was greater for nonsense signs than BSL. This stimulus effect was also greater in deaf than hearing signers. Each data point is also displayed as a black dot. Error bars indicate standard errors.

All statistical details are reported in Table 4. The parameter estimates for the ROIs are plotted in Fig. 7. In order to compare with the findings reported by Mayberry et al. (2011), within the left and right ROIs we first report the results of the analyses in deaf signers only and then compare deaf and hearing groups.

**Table 4:** Statistical details for the posterior STC ROI analyses. * ​= ​degrees of freedom were corrected following a violation of the equal variance (t-tests) or sphericity (ANOVA) assumption. ** ​= ​significant at *p* ​< ​.05 FWE corrected. AoA ​= ​age of acquisition.

		Left STC ROI				Right STC ROI			
Deaf signers		df	F	p	ω^2^	df	F	p	ω^2^
AoA		1,24	3.994	0.057**	0.103	1,24	0.152	0.700	0.000
Stimulus type		1,24	33.185	<.001**	0.509	1,24	8.118	0.009	0.213
AoA * Stimulus type		1,24	5.027	0.034**	0.064	1,24	0.290	0.595	0.000

		*df*	*t*	*p*	*d*				
Deaf early ​> ​deaf late for BSL		21.30*	2.730	0.012**	1.031				
Deaf early ​> ​deaf late for nonsense signs		24	1.101	0.282	0.437				

*Deaf signers and hearing signers*		*df*	*F*	*p*	*ω*^*2*^	*df*	*F*	*p*	*ω*^*2*^
Stimulus type		1,48	70.828	<.001 **	0.556	1,48	1.127	0.294	0.002
Stimulus type *Hearing status		1,48	1.402	0.242	0.003	1,48	10.715	0.002**	0.159
Stimulus type * AoA		1,48	0.980	0.327	0.000	1,48	0.100	0.753	0.000
Hearing status		1,48	0.267	0.608	0.000	1,48	10.528	0.002**	0.159
AoA		1,48	0.535	0.468	0.000	1,48	0.028	0.868	0.000
AoA *Hearing status		1,48	4.739	0.034 **	0.069	1,48	0.278	0.600	0.000
AoA *Hearing status *Stimulus type		1,48	3.330	0.074	0.019	1,48	0.252	0.618	0.000

		*df*	*t*	*p*	*d*				
Deaf early ​> ​deaf late		21.64*	2.200	0.039**	0.832				
Hearing early ​> ​hearing late		24	−1.053	0.303	−0.414				

						*df*	*t*	*p*	*d*
Deaf ​> ​Hearing for BSL						38.00*	2.285	0.028**	0.634
Deaf ​> ​Hearing for nonsense signs						38.50*	4.109	<.001**	1.140

**Left posterior STC ROI:** Deaf participants who had learnt BSL early showed greater activation in response to BSL in the left posterior STC ROI compared to deaf participants who learnt BSL late. This effect was not observed for strings of nonsense signs. This difference was reflected in the significant age of BSL acquisition by stimulus type interaction in deaf signers. In hearing signers, there was no effect of age of acquisition for BSL or nonsense signs in this ROI. There was no main effect of hearing status. Critically, the two-way interaction between age of acquisition by hearing status interaction was significant. Activation was greater in deaf early signers than deaf late signers, while there was no difference in activation between hearing early and hearing late signers. Furthermore, responses in early deaf signers did not differ from those of the two hearing groups. The three-way interaction (age of acquisition by hearing status by stimulus type) was not significant. A significant effect of stimulus type indicated that the left posterior STC ROI was significantly more activated by BSL than nonsense signs across both groups, but there was no hearing status by stimulus type interaction.

**Right posterior STC ROI:** There was no significant effect of age of acquisition in the right posterior STC ROI in deaf participants during either condition or across conditions. However, there were main effects of hearing status (greater activation for deaf than hearing signers) and stimulus type (greater activation for strings of nonsense signs than BSL). There was also a significant hearing status by stimulus type interaction such that the effect of hearing status was significant for nonsense signs but not for BSL (with Bonferroni corrections).

In summary, we observed two age of BSL acquisition effects. The first was a main effect of age of BSL acquisition across task and hearing status in the occipital segment of the left intraparietal sulcus, where activation was greater in late than early signers. The second was only in deaf signers in the left posterior STC region of interest, where activation was greater in deaf early signers than deaf late signers for BSL but not nonsense signs. There was no effect of age of sign language in hearing signers in this region and no main effect of hearing status. The middle STC, that showed a significant bilateral main effect of hearing status (deaf ​> ​hearing; see Fig. 4), did not show an interaction with age of acquisition.

## 5 Discussion

Deaf and hearing people who learn a sign language after early childhood differ greatly in their early language experience. Hearing late signers learn a sign language upon the basis of a robust first spoken language. In contrast, the majority of deaf people who learn a sign language later in life have, by definition, had impoverished access to an early spoken language. The degree of access to spoken language can depend on many audiological factors. Degree of progress with spoken language acquisition depends on a range of social and cognitive factors, many of which are yet to be clearly determined. Thus, deaf late signers provide a unique perspective on the impact of different degrees of impoverished early language exposure on the neurobiology of language: insights that cannot be gained from research with hearing people alone. The very few previous group studies that have examined the influence of age of sign language acquisition on the neural systems supporting language processing have examined either deaf signers (Mayberry et al., 2011; MacSweeney et al., 2008) or hearing signers (Newman et al., 2002). These studies suggest that effects of age of sign language acquisition are very different in deaf and hearing signers. Here, we tested hearing and deaf, from birth, early and late signers on a BSL sentence processing task. Our aim was to determine whether differences in early language exposure between deaf and hearing late signers had an impact on the neural systems supporting processing of language learnt later in life (BSL).

We found a main effect of age of sign language acquisition (late ​> ​early) in the occipital segment of the left intraparietal sulcus. This effect did not differ between deaf and hearing participants. Surprisingly, despite differences in early language experience, differential effects of age of sign language acquisition between deaf and hearing signers were limited to the left STC ROI, where activation during BSL perception was greater for early than late signers in deaf participants but not in hearing participants. That more extensive group differences were not found may be due to the relatively good English language skills and BSL skills of the deaf late signers who were recruited in the current study in order to minimise differences between deaf early and late signers. Below we consider each of these key findings.

### 5.1 Greater activation in late than early signers, regardless of hearing status

Late signers, both deaf and hearing, recruited the occipital segment of the left intraparietal sulcus more than early signers. Similarly, Mayberry et al. (2011) reported a positive correlation in deaf signers between age of ASL acquisition and activation in the left middle occipital gyrus (lateral to the occipital segment of the intraparietal sulcus) and also in the left lingual gyrus. In these regions, there was greater activation for those with later than earlier age of ASL acquisition during grammatical and phonemic judgements of ASL sentences. Mayberry et al. (2011) interpret this pattern as reflecting greater reliance on a shallow/perceptual level of language processing in deaf late than early signers. They further argue that this pattern does not resemble that seen in typical ‘L2’ learners, but is likely to be due to the poor early language experience of deaf late learners of sign language.

Our study tests this possibility by contrasting deaf and hearing early and late signers. There was no evidence that the effect of sign language acquisition (late ​> ​early) we identified, in the occipital segment of the intraparietal sulcus, was influenced by hearing status or stimulus type. Therefore, the effect we observed in occipital regions could not be explained by early language experience because the same effect (late ​> ​early) was observed for deaf late signers (with impoverished L1) and hearing late signers (with a robust L1), who are typical L2 learners. Our data therefore suggest that greater activation in late than early signers in the occipital segment of the intraparietal sulcus, should be interpreted as reflecting processes common, in both deaf and hearing late signers, to both BSL sentence and nonsense sign perception.

One possible explanation is that the late and early signing groups differed in sign language proficiency. Some support for this explanation comes from the responses to the in-scanner task: early signers were quicker to respond than late signers. However, the data contributing to these analyses were sparse as responses were only required to target trials, which made up only 20% of the total number. Furthermore, the groups did not differ in accuracy on this task. Participants in the current study were also tested on an off-line test of BSL grammaticality judgment (Cormier et al., 2012 ). There were no differences between the four groups on this test. Thus, on the basis of the assessments used, early and late signers in the current study did not appear to differ greatly in sign language proficiency and therefore sign language proficiency is unlikely to fully explain the pattern observed.

In line with the general proposal of Mayberry et al. (2011) it is likely that late sign language learners, both deaf and hearing, may rely on shallower, more visual, processing resources when perceiving strings of nonsense signs and sign language than early learners. Further studies are necessary to determine what aspects of these stimuli are being processed in these regions. For example, the greater activation in late learners could reflect general human action processing (see e.g., Corina et al., 2007) or potentially higher-level processing, given that the nonsense signs in the current study were phonotactically legal.

Other regions that have previously been shown to be sensitive to effects of age of sign language acquisition by Mayberry et al. (2011), MacSweeney et al. (2008) and Newman et al. (2002) (see Introduction) were not identified in the current study. Discrepancies across previous studies of deaf signers and of hearing signers, and also the current study, highlight that any age of acquisition effects are likely to be dependent on tasks, stimuli and a range of participant factors, including both sign language and spoken language proficiency. In the current study, deaf late signers had good BSL proficiency and also good English proficiency, as measured by a vocabulary test and reading comprehension test. Reading level was significantly poorer than that of hearing late signers, but it was good (equivalent to approx 17yrs). In contrast, in the Mayberry et al. (2011) study there was a negative correlation between self-rated ASL skills and age of acquisition in the deaf signers. No spoken language assessments were reported. Such individual differences are likely to have an important impact on any influence of age of sign language acquisition. A complete picture of the impact of profound congenital deafness on the neural systems supporting language in non-native signers will only be gained by examining the full range of language outcomes in deaf people: from those brought up as language isolates (e.g., Ramirez et al., 2014), often in developing countries, to those included in the current study who, despite impoverished early access to spoken language, have established relatively good spoken language and, later in life, sign language skills.

### 5.2 Greater activation in early than late signers in deaf participants, only in left posterior STC

One aim of the study was to determine whether activation in the STCs in response to sign language input, was influenced by age of sign language acquisition in deaf signers. Mayberry et al. (2011) reported a negative correlation between age of ASL acquisition and activation in the bilateral posterior STCs in deaf signers (greater in early than late signers) during an ASL sentence grammaticality judgement task. Using this region as an ROI in left STC our analyses replicated the results of Mayberry et al. (2011) in deaf signers. During BSL perception activation was greater in deaf early than late signers in the left posterior STC. Our design furthers our understanding of this effect in the left posterior STC in the following ways. First, the effect of sign language acquisition (early ​> ​late) in this region is observed only in deaf signers. No effect of age of sign language acquisition was observed in hearing signers. Second, and importantly, there was no main effect of hearing status in the left posterior STC ROI. Responses, during BSL perception, in the left STC ROI were equivalent in hearing signers (early and late) and deaf early signers: the three groups who had robust early language experience. Third, the effect in the left STC ROI in deaf signers (early ​> ​late) was significant for BSL but not phonotactically legal nonsense signs. Together these findings suggest that the left posterior STC ROI identified by Mayberry et al. (2011) and investigated here, is responsive to signed input in both deaf and hearing signers. However, the impact of late language exposure is different in these two groups in this region. Only in deaf late signers, with impoverished early spoken language exposure, is there a long-lasting effect on the neural processing of language.

In a recent study, we reported extensive effects of hearing status (deaf ​> ​hearing) in the *middle STCs* but no influence of age of sign language acquisition (Twomey et al., 2017). In the Twomey et al. (2017) study participants were asked to make BSL metalinguistic judgements about pictures regarding semantics and BSL phonology, as opposed to perceiving sign language stimuli as in the present design. Post-hoc analyses of the Twomey et al. (2017) dataset, for the purposes of the current paper, using the STC ROI derived from Mayberry et al. (2011) confirmed that there was no interaction in left or right STC ROIs between hearing status and age of acquisition (Z ​< ​1.77). This suggests that effects of age of sign language acquisition in this region may only be observed during perception of sign stimuli. Further studies are needed to determine what specific stimulus characteristics are necessary to observe an effect of age of sign language acquisition in the left posterior STC.

In contrast to the left STC ROI, the right posterior STC showed no significant effect of age of sign language acquisition in deaf or hearing signers. There was a significant main effect of hearing status (deaf ​> ​hearing) and also a hearing status by stimulus type interaction, indicating that the effect of hearing status was significant for nonsense signs but not for BSL. Given that an effect of age of sign language acquisition was observed in the left posterior STC ROI for BSL stimuli but not nonsense sign sequences and the strong left lateralised processing of signed stimuli (e.g. MacSweeney et al., 2008), it is perhaps not surprising that the right STC is not engaged differently in deaf early and late signers, matched on a test of BSL grammaticality judgement, during sign language comprehension. This finding is contrary to the findings of Mayberry et al. (2011) who reported a negative relationship between age of ASL acquisition and activation in the right as well as left posterior STC. The effect in right STC in Mayberry et al.’s (2011) study may reflect the effect of sign language proficiency. Mayberry et al. (2011) reported a trend towards a negative correlation between age of ASL acquisition and self-reported ASL comprehension skills. In contrast, in the current study performance on a BSL grammaticality test (Cormier et al., 2012) was matched across groups. Therefore, one possibility is that ASL proficiency, rather than age of ASL acquisition, was positively correlated with the right STC activation in the Mayberry et al. (2011) study.

### 5.3 Hearing status effects in bilateral STCs

Replicating numerous previous studies, main effects of hearing status (deaf ​> ​hearing) were found in an extensive area of the STCs bilaterally, with the strongest effects in the middle STCs. The location of these effects shared very little overlap with the posterior STC ROIs (one voxel in the left ROI). The responses in deaf signers were of activation, whereas those in hearing signers were mostly of deactivation. There was no evidence that hearing status effects were stronger for BSL than nonsense signs at the whole brain level and no influence of age of sign language acquisition. Therefore, in contrast to the age of acquisition effect in left posterior STC described above, the increased middle STC activation in deaf compared to hearing signers in the current study is likely to be due to processes common to both BSL sentences and nonsense sign sequences.

Hearing status effects (deaf ​> ​hearing) in the STCs were extensive and included a region of primary auditory cortex in Heschl’s gyrus – here mapped only at the group level, not the individual participant level (see Cardin et al., 2016). However, activation in Heschl’s gyrus in deaf early signers and deaf late signers was no different from the baseline (Table 3). In contrast, both hearing early and hearing late signers deactivated the left Heschl’s gyrus significantly at the *p* ​< ​.001 uncorrected level. Therefore, the significant difference between deaf signers and hearing signers in the left Heschl’s gyrus was due to hearing signers suppressing task-irrelevant auditory activity, rather than deaf signers recruiting this region during the visual tasks. This interpretation supports that of our previous study (Twomey et al., 2017) and that of Cardin et al. (2016; see also Karns et al., 2012) and again highlights the importance of examining the direction of responses in hearing participants when interpreting effects of hearing status.

### 5.4 Potential implications of STC findings

The finding of an age of sign language acquisition effect (early ​> ​late) in left posterior STC in deaf signers has potentially important implications for advice given to parents of deaf children. In some situations hearing parents of deaf children have been advised to not expose their child to sign language for fear of ‘take over’ of parts of auditory cortex by visual input (see Campbell et al., 2014 for review). Here we did find that early deaf signers showed greater responses to BSL sentences than nonsense signs in the posterior part of the left STC, whereas the deaf late signers did not show this difference. Critically however the responses of deaf early signers did not differ to that of hearing participants. Rather, deaf late signers, with impoverished early language experience, showed reduced activation for BSL compared to the other three groups. Our data therefore suggest that impoverished early exposure to language results in subtle differences in how the neural system in the left posterior STC is recruited to process a language learnt later in life. This neural difference was observed despite the fact that deaf late signers had high BSL proficiency. The data therefore suggest that robust early language experience, whether signed or spoken, is necessary for this region to show a ‘native-like’ response to a later learnt language.

### 5.5 Differential effects of stimulus type for deaf and hearing participants in left precentral gyrus

We also found significant interaction between hearing status and stimulus type in the left precentral gyrus. The significant interaction was driven by the opposite pattern of activation in deaf (nonsense signs ​> ​BSL) and hearing signers (BSL ​> ​nonsense signs). MacSweeney et al. (2004) reported greater activation in hearing signers compared to deaf signers in response to BSL but not to nonsense signs in the left posterior inferior frontal gyrus, which is close to the location of the significant interaction in the current study. Here, activation was also numerically greater in hearing than deaf signers for BSL but not for nonsense signs, although this difference was not significant. Although this effect does not involve age of sign language acquisition, the focus of the current paper, we report the findings in full here in order to provide input to future studies in the field. Further studies are needed to investigate the possible causes of these intriguing potential differences in recruitment of the left prefrontal cortex between deaf and hearing signers during sign language perception.

## 6 Conclusion

We investigated whether the effects of age of sign language acquisition differed between deaf and hearing signers, who differ in early language exposure. There are two main findings. First, we found an effect of age of sign language acquisition that was common to both deaf and hearing signers in the occipital segment of left intraparietal sulcus where activation was greater for late than early signers. This effect may reflect greater reliance on shallower, visual, processing resources when perceiving sign language and nonsense signs in late learners, regardless of hearing status. Second, we found an effect of age of sign language acquisition that was specific to deaf signers in the left posterior STC where activation in response to signed sentences was greater in deaf early signers than deaf late signers. This effect was not observed for nonsense sign sequences and not in hearing signers, whose responses in this region were similar to that of deaf early signers. No effect of age of sign language acquisition was found in the middle STCs. Our findings suggest that any effect of age of acquisition in the left STC is only likely to be observed in the *posterior* STC: a multisensory region in hearing people, as demonstrated here by the responses to sign stimuli in hearing signers. These data lend further support to the argument that robust early language experience, whether signed or spoken, is necessary for left posterior STC to show a ‘native-like’ response to a later learnt language.

## Author contributions section

Ref: NIMG 19-1410.

Tae Twomey: Data analysis; Manuscript writing.

Cathy J. Price: Data analysis supervision; Manuscript writing.

Dafydd Waters: Methodology; Data Collection.

Mairéad MacSweeney: Conceptualization; Data collection; Supervision; Manuscript writing.

## Declaration of competing interest

None.
